# RHOB Regulates Apoptosis of Granulosa Cells in Muscovy Duck Follicles via Mitochondrial Pathway

**DOI:** 10.3390/ani16111711

**Published:** 2026-06-03

**Authors:** Yuexia Liu, Xin Wang, Leyong Li, Yaping Zhang, Senyang Lian, Xu Wu

**Affiliations:** Engineering Research Center for Animal Breeding and Sustainable Production, College of Animal Sciences, Fujian Agriculture and Forestry University, Fuzhou 350002, China; liuyuexia1228@163.com (Y.L.); wangxin@163.com (X.W.); lileyong202509@163.com (L.L.); ypzhang0725@163.com (Y.Z.); liansenyang@163.com (S.L.)

**Keywords:** Muscovy duck, follicular granulosa cells, apoptosis, mitochondria

## Abstract

Muscovy ducks (*Cairina moschata*) exhibit strong nesting tendencies, which result in reduced egg-laying performance. The research team previously identified differential expression of the Ras homolog family member B (RHOB) gene in the ovaries of Muscovy duck during the nesting and laying periods through RNA-seq and quantitative real-time polymerase chain reaction (qPCR) analysis. This finding suggested that RHOB may be associated with nesting behavior in Muscovy ducks. Previous studies have demonstrated that the nesting behavior of Muscovy ducks is closely associated with the proliferation and apoptosis of their ovarian granulosa cells. It is speculated that RHOB may be involved in the proliferation and apoptosis of Muscovy duck ovarian granulosa cells. This study employed qPCR, immunofluorescence staining, live-cell Caspase3 activity and mitochondrial membrane potential assays, reactive oxygen species (ROS) staining, 5-Ethynyl-2′-deoxyuridine (EdU) staining, cell cycle analysis, cell apoptosis detection and cell counting kit-8 (CCK-8) assays. Our results showed that RHOB inhibited granulosa cell apoptosis and promoted granulosa cell proliferation. Similarly, in a granulosa cell apoptosis model, RHOB was also found to inhibit apoptosis in Muscovy duck granulosa cells. Further studies revealed that RHOB regulates mitochondrial function in granulosa cells. The combined experimental results indicate that RHOB regulates granulosa cell apoptosis in Muscovy duck follicles via the mitochondrial apoptosis pathway. These findings provide an experimental basis and theoretical foundation for the selective breeding of desirable traits in Muscovy ducks, such as low nesting behavior and high egg production.

## Simple Summary

Muscovy ducks (*Cairina moschata*) exhibit strong nesting tendencies, which result in reduced egg-laying performance. Previous studies by our group have shown that the Ras homolog family member B (RHOB) gene is differentially expressed in the ovaries tissue of Muscovy ducks during the nesting and laying periods, and is associated with nesting behavior of Muscovy ducks. This study experimentally demonstratedthat RHOB regulates granulosa cell apoptosis in Muscovy duck follicles via the mitochondrial apoptosis pathway. These findings provide an experimental basis and theoretical foundation for the selective breeding of desirable traits in Muscovy ducks, such as low nesting behavior and high egg production.

## 1. Introduction

Muscovy ducks, also known as *Cairina moschata*, lay egg rich in nutrients that can boost human immunity and promote health. However, their strong nesting instinct significantly reduced laying, leading to lower laying rates or even cessation of egg-laying, which severely limited the economic viability of Muscovy duck farming. Therefore, elucidating the molecular mechanisms underlying this nesting behavior is crucial for enhancing the economic efficiency of Muscovy duck farming.

As one of the primary reproductive organs, the ovary plays a crucial role in the production and reproduction of poultry. The follicle, being the most important part of the ovary, directly influences egg production [1]. Abnormal proliferation or apoptosis of granulosa cells can lead to impaired follicular development, defective oocyte maturation, and chromosomal abnormalities in oocytes. In turn, reduced oocyte quality negatively impacts reproductive performance [2]. During the nesting period, Muscovy ducks primarily exhibited follicular atresia and massive granulosa cell apoptosis [1,3,4]. Therefore, exploring the genetic and molecular mechanisms regulating follicular development and granulosa cell proliferation and apoptosis in Muscovy ducks, holds significant practical importance for reducing nest-sitting behavior and improving egg-laying performance in Muscovy ducks.

The proliferation and apoptosis of granulosa cells were closely related to ovarian function and follicular development. Studies have shown that mitochondrial dysfunction reduces granulosa cell survival and promotes apoptosis, impairing granulosa cell function and thereby leading to a decline in ovarian reserve [5,6]. At the same time, alterations in the autophagy and apoptosis processes of granulosa cells in the chicken ovary were found to affect follicular atresia in the chicken ovary [7]. Furthermore, it was found that apoptosis and inflammatory responses in ovarian granulosa cells lead to follicular atresia [8,9]. Research has also found that neuropeptide Y regulated granulosa cells proliferation and apoptosis in manner dependent on the stage of follicular development [10]. These studies collectively indicated that the proliferation and apoptosis of granulosa cells were closely associated with ovarian function and follicular atresia [11,12]. Therefore, investigating the molecular mechanisms regulating granulosa cell proliferation and apoptosis in Muscovy duck follicles is of great significance for promoting follicular development and enhancing egg production.

Ras homolog family member B (RHOB) was a well-known tumor suppressor gene. The RHO GTPase family possesses the ability to bind both GTP and GDP. When bound to GTP, it is in an active state, whereas binding to GDP places it in an inactive state. This affects vital cellular activities such as migration, differentiation, growth, and apoptosis [13,14]. The RHO subfamily comprises three proteins: RHOA, RHOB, and RHOC. RHOB has been extensively studied as a tumor suppressor gene [14,15]. RHOB is closely associated with apoptosis. Studies indicated that the TOX high mobility group box family member 3 (TOX3) molecule in colorectal cancer cells downregulates RHOB through activation of the mitogen-activated protein kinase (MAPK) signaling pathway, thereby regulating proliferation and apoptosis in colorectal cancer [16]. RHOB-GTP regulated human epidermal growth factor receptor 2 (HER2) and epidermal growth factor receptor (EGFR) signaling pathways in breast cancer cells by interacting with the scaffolding protein connector enhancer of kinase suppressor of Ras 1 (CNKSR1), thereby influencing breast cancer proliferation [17]. RHOB participated in the regulation of Caspase3-dependent apoptosis, reactive oxygen species (ROS) production, and autophagy flux through molecular interactions with cysteine-specific aspartate protease 3 (Caspase3) [18]. It is evident that RHOB is closely associated with cellular proliferation, cell cycle progression, and apoptosis.

To date, no studies have been reported on the role of RHOB in the granulosa cells of Muscovy duck follicles. The aim of this study is to investigate the regulatory role of RHOB in the proliferation and apoptosis of Muscovy duck granulosa cells.

## 2. Materials and Methods

### 2.1. Isolate and Identify Granulosa Cells from Muscovy Duck Follicles In Vitro

We collected the entire ovaries of Muscovy ducks and selected the small yellow follicles. Removed the outer connective tissue layer from the small yellow follicles, then separated and purified the granulosa cells from Muscovy duck follicles using trypsin digestion combined with differential adhesion. The isolated and purified follicular granulosa cells from Muscovy duck laying hens were cultured and passaged. Then, the granulosa cells in Muscovy duck follicles were identified using techniques such as qPCR, Western blotting, and immunofluorescence staining for follicle-stimulating hormone receptor (FSHR) protein.

### 2.2. Cell Processing

The isolated and purified muscovy duck follicular granulosa cells were cultured and seeded into 6-well plates. Next, the RHOB (XM_038177654.2) siRNA construct (Si-*Rhob*) and the overexpression vector (pC-*Rhob*) were constructed, and the follicular granulosa cells were transfected with 2 μg each of pC3.1, pC-*Rhob*, Si-Control, and Si-*Rhob*. Cells were then cultured for 24 or 48 h, and collectedfor further processing.

### 2.3. Real-Time Quantitative PCR (qPCR) Analysis

Total RNA was extracted from various tissues or granulosa cells of Muscovy duck follicles. A total of 500 ng RNA was reverse-transcribed using and the SuperRT III All-in-one RT Mix with gDNA Remover (Biosharp, Shanghai, China). All primers were synthesized by Qingke Biotechnology (Qingke Biology, Beijing, China). Quantitative PCRwas performed in a 10 μL reaction system containing specific primers and SYBR Green Master Mix (Biosharp, Shanghai, China). The names and sequence information of the primers are shown in Table 1.

### 2.4. Immunofluorescence Assay

After fixing follicular cells with 4% paraformaldehyde (SolaBio, Beijing, China), permeabilizing with 0.1% Triton X-100 (ThermoFisher, Shanghai, China), and then blocking with 5% bovine serum albumin (BSA, Yeasen, Shanghai, China) at room temperature for 1 h, the cells were incubated with anti-FSHR antibody at room temperature for 2 h, followed by incubation with fluorescent secondary antibody at room temperature for 1 h. Finally, DAPI (MedChemExpress, Shanghai, China) staining was performed for 5 min and the cells were observed and photographed using a fluorescence microscope.

### 2.5. Detection of Caspase3 Activity and Mitochondrial Membrane Potential in Living Cells

Treated Muscovy duck granulosa cells were stained using the Live Cell Caspase3 Activity and Mitochondrial Membrane Potential Detection Kit (Beyotime, Shanghai, China) according to the kit instructions. Following staining, observations and photographs were taken using a fluorescence microscope.

### 2.6. ROS Staining

The treated Muscovy duck granulosa cells were stained using the ROS staining kit (Beyotime, Shanghai, China) according to the kit instructions. Following staining, the samples were observed and photographed using a fluorescence microscop.

### 2.7. EdU Staining

The treated Muscovy duck follicular granulosa cells were stained using the EdU staining kit (Beyotime, Shanghai, China). The procedure was performed according to the kit instructions. Following staining, the samples were observed and photographed using a fluorescence microscope.

### 2.8. Cell Apoptosis Detection

Detect apoptosis following interference and overexpression of RHOB using the Annexin V-APC/PI Apoptosis Detection Kit (UE, Shanghai, China). Follow the kit instructions, resuspend the cells in 400 μL of 1× Annexin V Binding Buffer and immediately analyze apoptosis by flow cytometry.

### 2.9. Cell Cycle Detection

Detect cell cycle status after interference and overexpression of RHOB using the Cell Cycle Assay Kit (SolaBio, Beijing, China), following the kit instructions. After PI staining, incubate at 4 °C in the dark for 30 min. Detect the intensity of red fluorescence at an excitation wavelength of 488 nm for cells in each group using a flow cytometer, while simultaneously measuring light scattering.

### 2.10. CCK8 Assay

The Super-Enhanced Cell Counting Kit-8 (Super-Enhanced CCK-8 Kit) (Beyotime, Shanghai, China) was used to detect cell viability after interference and overexpression of RHOB. Following the kit instructions, cell absorbance values were measured using a microplate reader at 0.5 h, 1 h, 1.5 h, 2 h, and 3 h after adding the CCK-8 staining solution.

### 2.11. Statistical Analysis

All data were analyzed for significant differences using one-way ANOVA and two-way ANOVA. Fisher’s least significant difference (LSD) was used for comparison of individual means. Data are expressed as mean ± standard deviation, and *p* < 0.05 was considered significant.

## 3. Results

### 3.1. RHOB Inhibited Apoptosis in Granulosa Cells of Muscovy Duck Follicles

**Figure 1:**
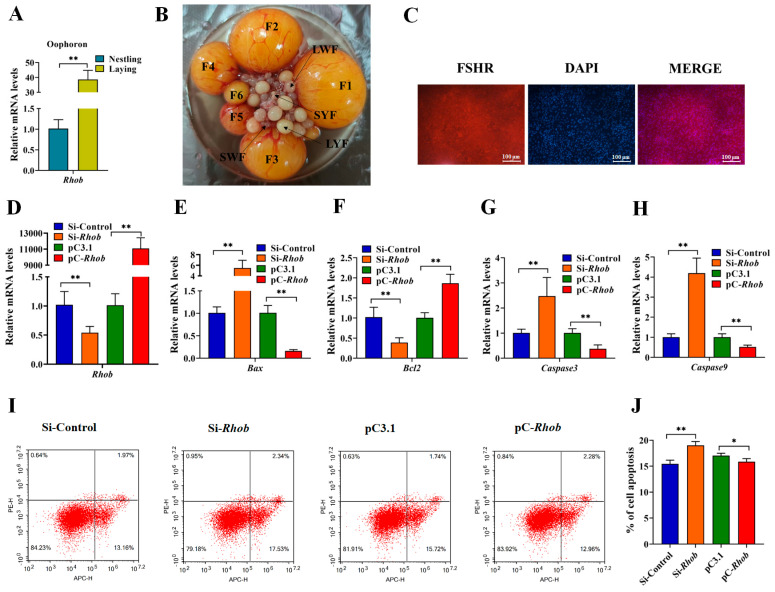
RHOB inhibited follicular granulosa cell apoptosis in Muscovy ducks. (**A**) Relative mRNA expression levels of RHOB in the oophoron of Muscovy ducks during the nesting and laying periods (n = 4). (**B**) Images of follicular stages in Muscovy ducks. (**C**) Immunofluorescence staining image of FSHR, scale bar: 100 μm. (**D**) RHOB expression efficiency assay (n = 4). (**E**–**H**) Relative mRNA expression levels of apoptosis-related genes following RHOB overexpression and knockdown (n = 4). (**I**,**J**) Flow cytometry analysis of apoptosis following RHOB overexpression and knockdown (n = 4). * indicates *p* < 0.05, ** indicates *p* < 0.01, n represents the number of technical repetitions.

This study first examined the expression of RHOB in ovarian tissues from Muscovy ducks during the nesting and laying periods. The results showed that RHOB was highly expressed in the ovaries of Muscovy ducks during the laying period (Figure 1A). To investigate the role of RHOB in the apoptosis of Muscovy duck follicular granulosa cells, whole ovaries were first collected (Figure 1B). Follicular granulosa cells were then isolated and cultured in vitro, and identified via FSHR immunofluorescence staining. The results confirmed that the isolated cells were follicular granulosa cells (Figure 1C). Next, we constructed the overexpression vectors and interference fragments of RHOB and transfected them into Muscovy duck granulosa cells, revealing that RHOB overexpression resulted in a relative increase in RHOB expression of more than 10,000-fold. After interfering with RHOB, expression was reduced by 50%, with both changes being highly significant (Figure 1D), confirming their suitability for subsequent experiments. Furthermore, it was revealed that RHOB significantly suppressed the expression of pro-apoptotic genes BAX, Caspase3 and Caspase9 while promoting the anti-apoptotic genes BCL2 (Figure 1E–H), preliminarily indicating that RHOB inhibited apoptosis in Muscovy duck follicular granulosa cells. Finally, flow cytometry analysis revealed that RHOB inhibited apoptosis in Muscovy duck follicular granulosa cells (Figure 1I,J), consistent with previous findings. Collectively, these results demonstrate that RHOB suppresses apoptosis in Muscovy duck follicular granulosa cells.

### 3.2. RHOB Promoted Follicular Granulosa Cell Proliferation in Muscovy Duck

**Figure 2:**
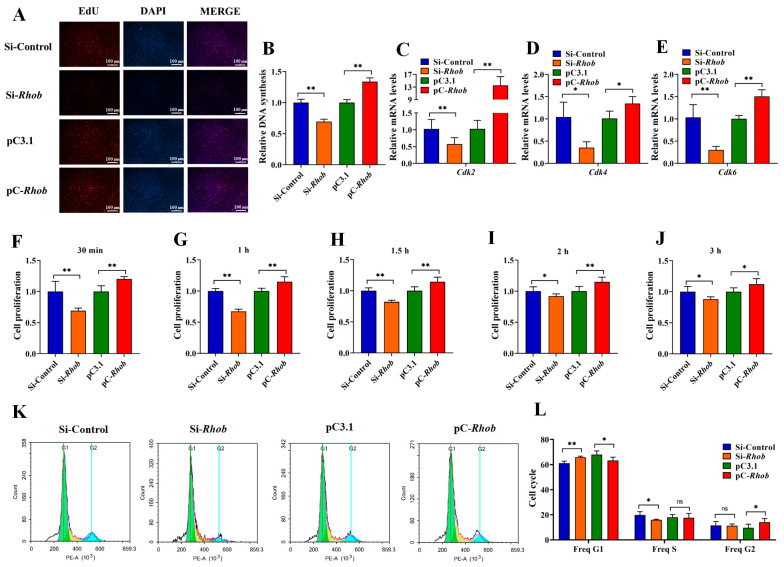
RHOB promoted granulosa cell proliferation in Muscovy duck follicles. (**A**,**B**) EdU staining and fluorescence quantitative analysis, scale bar: 100 μm (n = 3). (**C**–**E**) Relative mRNA levels of cell cycle-related genes after RHOB overexpression and knockdown (n = 4). (**F**–**J**) CCK8 assay at different time points after RHOB overexpression and interference (n = 4). (**K**,**L**) Flow cytometry analysis of cell cycle after RHOB overexpression and interference (n = 4). ns indicates *p* > 0.05, * indicates *p* < 0.05, ** indicates *p* < 0.01, n represents the number of technical repetitions.

Previous studies revealed that RHOB inhibits apoptosis in Muscovy duck follicular granulosa cells. To further investigate the effects of RHOB on these cells, its role in promoting proliferation was subsequently examined. EdU staining revealed that RHOB significantly promoted the proliferation of granulosa cells in Muscovy duck follicles (Figure 2A,B). It was revealed that RHOB significantly promoted the expression of genes CDK2, CDK4 and CDK6 associated with cell proliferation (Figure 2C–E). Cell viability was assessed using the CCK-8 assay, revealing that RHOB promotes cell viability (Figure 2F–J). Finally, flow cytometry analysis revealed that RHOB inhibits G1 phase arrest in Muscovy duck follicular granulosa cells (Figure 2K,L); in combination with the findings described in Section 3.1, these results suggest that RHOB may play a role in suppressing apoptosis. Collectively, these findings demonstrate that RHOB promotes proliferation of Muscovy duck follicular granulosa cells.

### 3.3. Establish an Apoptotic Cell Model of Granulosa Cells in Muscovy Duck Follicles

**Figure 3:**
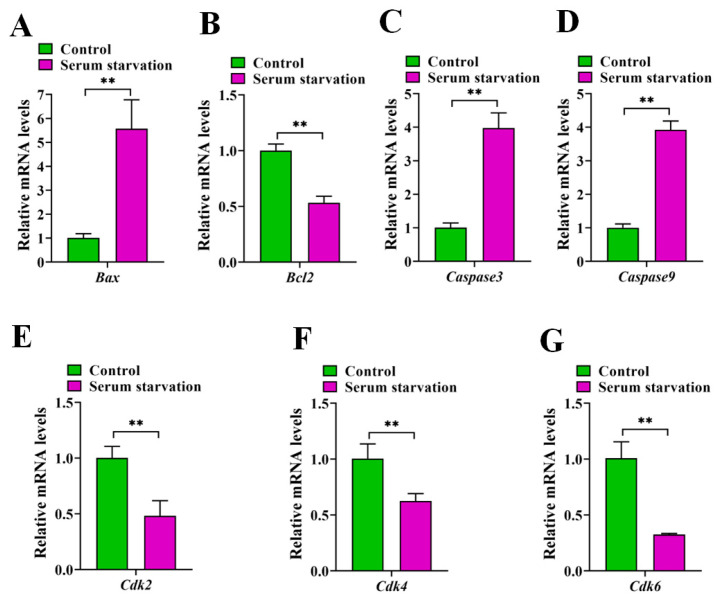
Validation of the apoptosis model in granulosa cells of Muscovy duck follicles. (**A**–**D**) Relative mRNA levels of apoptosis-related genes following serum starvation (n = 4). (**E**–**G**) Relative mRNA levels of cycle-related genes after serum starvation (n = 4). ** indicates *p* < 0.01, n represents the number of technical repetitions.

To further investigate the role of RHOB, a serum-deprivation model of granulosa cell apoptosis was established and validated. The detection results revealed that serum-deprived cells exhibited significantly enhanced expression of pro-apoptotic genes BAX, Caspase3 and Caspase9 (Figure 3A–D) and suppressed expression of cell cycle-related genes CDK2, CDK4 and CDK6 (Figure 3E–G) compared to the control group. The above results indicate that the apoptosis model has been successfully established.

### 3.4. Overexpression of RHOB Alleviated Serum-Induced Apoptosis in Granulosa Cells of Muscovy Duck Follicles

**Figure 4:**
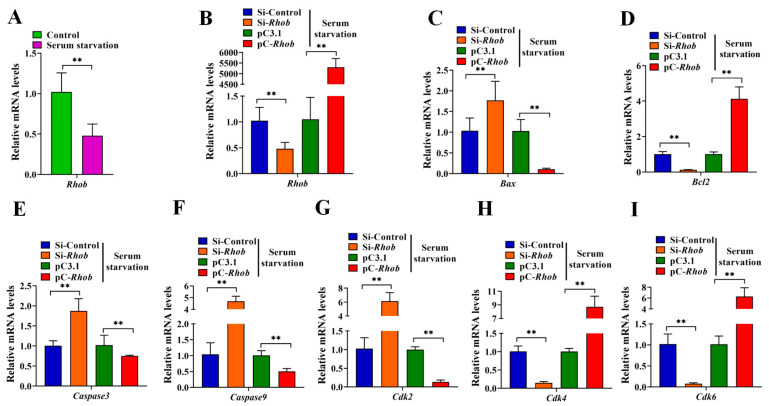
Overexpression of RHOB alleviated serum-induced apoptosis in granulosa cells of Muscovy duck follicles. (**A**) Relative mRNA expression levels of RHOB following serum starvation (n = 4). (**B**) Serum starvation followed by overexpression and interference of RHOB, showing the efficiency of RHOB expression (n = 4). (**C**–**F**) Relative mRNA expression levels of apoptosis-related genes following serum starvation and overexpression or knockdown of RHOB (n = 4). (**G**–**I**) Relative mRNA levels of cell cycle-related genes following serum starvation and overexpression or knockdown of RHOB (n = 4). ** indicates *p* < 0.01, n represents the number of technical repetitions.

Further detection revealed a highly significant reduction in RHOB following serum starvation (Figure 4A), consistent with RHOB’s role in inhibiting apoptosis. Following serum-deprivation treatment of cells, overexpression and knockdown of RHOB were detected, revealing that the expression level of RHOB increased more than 5000-fold following overexpression, while RHOB expression was reduced by 50% following si-RHOB treatment, both changes were highly significant (Figure 4B). Further analysis revealed that, following serum-deprivation treatment, RHOB suppressed the expression of apoptosis-related genes BAX, Caspase-3, and Caspase-9, (Figure 4C–F), while promoting the expression of cell proliferation and cell cycle-related genes CDK2, CDK4, and CDK6 (Figure 4G–I). The above findings indicate that overexpression of RHOB alleviates serum-induced apoptosis in granulosa cells of Muscovy duck follicles.

### 3.5. RHOB Regulated Granulosa Cell Apoptosis in Muscovy Duck Follicles via Mitochondrial Pathway

**Figure 5:**
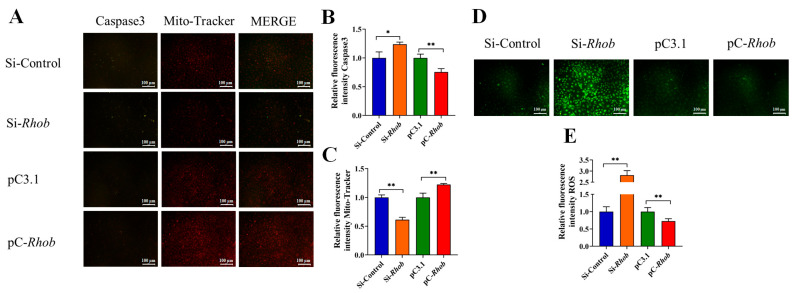
RHOB promoted mitochondrial activity in granulosa cells of Muscovy duck follicles. (**A**–**C**) Detection of Caspase3 and mitochondrial membrane potential in cells following RHOB overexpression and interference, along with fluorescence quantitative analysis, scale bar: 100 μm (n = 3). (**D**,**E**) ROS staining and fluorescence quantitative analysis after RHOB overexpression and interference, scale bar: 100 μm (n = 3). * indicates *p* < 0.05, ** indicates *p* < 0.01, n represents the number of technical repetitions.

Changes in mitochondrial function play a crucial role in the process of apoptosis. To investigate the role of mitochondria in RHOB-regulated proliferation and apoptosis of granulosa cells in Muscovy duck follicles, live-cell assays using a Caspase3 activity and mitochondrial membrane potential detection kit revealed that RHOB significantly suppressed Caspase3 levels while simultaneously promoting mitochondrial activity (Figure 5A–C). Furthermore, ROS staining revealed that RHOB significantly suppressed mitochondrial ROS production (Figure 5D,E). The combined results of the above studies indicate that RHOB significantly promotes mitochondrial activity in Muscovy duck follicular granulosa cells.

## 4. Discussion

The development of poultry follicles was influenced by oocytes, granulosa cells, and theca cells. Among these, granulosa cells primarily function to supply essential growth factors, nutrients, and metabolic intermediates to oocytes, while also secreting estrogen necessary for normal reproductive function [19]. Abnormal proliferation or apoptosis of granulosa cells can lead to impaired follicular development, defective oocyte maturation, and chromosomal abnormalities in oocytes. This results in reduced oocyte quality, negatively impacting reproductive performance. Furthermore, granulosa cell apoptosis can cause follicular atresia [20]. Within the atretic follicles, granulosa cells undergo apoptosis earlier than oocytes and theca cells. Massive apoptosis of granulosa cells was likely the key factor leading to follicular degeneration and atrophy [2]. When Muscovy ducks exhibited nesting behavior, it was accompanied by follicular atresia and granulosa cell apoptosis. This study also found differential expression of RHOB in ovarian tissues during the nesting and laying periods, suggesting a potential association between RHOB and nesting behavior in Muscovy ducks.

Apoptosis is also known as programmed cell death [21]. The RHOB molecule acts as a negative regulator of AKT’s antitumor activity in non-small cell lung cancer [22]. Downregulation of RHOB inhibited cancer cell proliferation, migration, and invasion while increasing apoptosis. Furthermore, downregulation of the RHOB gene enhances cisplatin-induced apoptosis [23]. Non-mutagenic stress-induced small G protein RHOB inhibited apoptosis and activates NF-κB [24]. Concurrently, RHOB knockdown partially counteracted the inhibitory effect of PITPNA-AS1 on ovarian cancer cell activity [25]. These studies collectively indicated that RHOB was associated with apoptosis. The results of this study showed that RHOB promoted the expression of proliferation-related genes, enhanced cell viability, and suppressed the expression of apoptosis-related genes. Flow cytometry analysis demonstrated that RHOB inhibited apoptosis in Muscovy duck follicular granulosa cells and promoted EdU activity, indicating that RHOB suppresses apoptosis in these cells. This finding is consistent with previously published research.

Among these, the intrinsic mitochondrial signaling pathway mediated by cysteine proteases (caspases) plays a crucial role in maintaining the balance between cell proliferation and cell death [26]. BCL2 was an anti-apoptotic protein gene located on the outer mitochondrial membrane, and the ratio of BAX to BCL2 determines cellular proliferation and apoptosis [27]. Under normal conditions, the expression of the pro-apoptotic protein BAX was suppressed by BCL2, which inhibited apoptosis in granular cells by regulating the release of cytochrome C and mitochondrial proteins during the apoptotic cascade. Upon receiving apoptotic signals, these proteins form heterodimers, releasing cytochrome C. This cytochrome C bound to apoptosis-activating factor 1 (APAF-1), forming apoptotic bodies that activated caspases and initiate the apoptotic mechanism [26,28]. Studies have also shown that granulosa cell apoptosis involves the mitochondrial pathway, the endoplasmic reticulum pathway, and the death receptor pathway, but is primarily mediated by the mitochondrial pathway through the caspase family [26]. This study detected that RHOB inhibits the relative expression levels of Caspase3, Caspase9, and BAX, and exhibits the same effects following serum starvation treatment. Further investigations revealed that RHOB significantly suppresses Caspase3 levels, promotes mitochondrial function, and concurrently inhibits reactive oxygen species (ROS) production. These findings indicate that RHOB regulates apoptosis in Muscovy duck follicular granulosa cells via the mitochondrial pathway.

## 5. Conclusions

This study’s results revealed that RHOB regulates the expression of apoptosis-related genes BAX, BCL2, Caspase3, Caspase9 and cell cycle-related genes CDK2, CDK4, and CDK6. Furthermore, it regulates apoptosis in granulosa cells of Muscovy duck follicles via the mitochondrial apoptosis pathway, thereby providing experimental evidence and a foundation for the selective breeding of high-quality Muscovy duck strains.

## Figures and Tables

**Table 1:** Names and sequence information of primer.

Primer	Sequence (5′—3′)
*Rhob*	F: GTAGTCGGAGATGACCGCTG
	R: CAGGCCTAACCACCGAGAAG
*Bax*	F: AAGTTCTCCCACATCTCGGC
	R: GGTTGATGGCGATGCACATT
*Bcl2*	F: TCTCGCAGAGGGGATACGAC
	R: CTGGTAGCGACGGGAGAAC
*Caspase* 3	F: AGGGGTGACAAGTGCAGAAG
	R: TTCCGCCAGGAGTAATAGCC
*Caspase* 9	F: GGATTGCGATTCACCCGAAG
	R: AGCGTTTCCACATACCACGA
*Cdk2*	F: GTCGTTTACAAGGCCCGGAA
	R: ACAGCTTGTTCTCCGTGTGG
*Cdk4*	F: CCCTTCTGCCTGGTAATCGG
	R: CGCTTGTAGGGGTTGAAGGT
*Cdk6*	F: CTCAGTGCCTGCATTTCTGCT
	R: GTGGTGCAATCGACACAAAGG
*Gapdh*-F	F: GAGGAGCTGCCCAGAACATT
	R: TGAAGTCGCAGGAGACAACC

## Data Availability

The datasets analyzed during the current study are available from the corresponding author upon reasonable request.
